# When Stress Becomes Human: The Joint Effect of Anthropomorphism and Generalized Trust on Stress Mindset

**DOI:** 10.3390/bs16081297

**Published:** 2026-07-30

**Authors:** Wenjian Fan, Qian Sun, Qianyun Gao, Lvmin Wu, Lin Liu

**Affiliations:** 1 College of Philosophy, Law & Political Science, Shanghai Normal University, Shanghai 200234, China; 2 Department of Psychology, Suzhou University of Science and Technology, Suzhou 215009, China; 3 School of Social Work and Health Management (Mental Health Education Center), Xihua University, Chengdu 610039, China; 0120220012@mail.xhu.edu.cn; 4 Centre for Mental Health Education, Neusoft Institute Guangdong, Foshan 528225, China; wulvmin@nuit.edu.cn

**Keywords:** stress mindset, anthropomorphism, generalized trust

## Abstract

Stress mindset—the extent to which individuals believe that stress has enhancing rather than debilitating consequences—is an important predictor of mental health, highlighting the need to identify factors that shape it. While previous research has shown that externally provided persuasive information about the beneficial consequences of stress fosters a more stress-is-enhancing mindset, little is known about whether and when individuals’ internal cognitive strategies can also foster this mindset. This research focuses on a specific internal cognitive strategy—anthropomorphism—and investigates whether it and generalized trust jointly shape individuals’ stress mindset. Two experimental studies were conducted. Study 1 shows that compared with non-anthropomorphism, anthropomorphism leads to a more stress-is-enhancing mindset among individuals with high generalized trust, but not among those with low generalized trust. Study 2 further demonstrates that this joint effect is mediated by enhanced perceived altruistic motivation. This research introduces stress as a new anthropomorphic entity and extends the stress mindset literature by showing that an internal cognitive strategy and a social belief jointly shape individuals’ stress mindset.

## 1. Introduction

Stress mindset refers to the extent to which individuals believe that stress has enhancing rather than debilitating consequences for their performance, health, and well-being (Crum et al., 2013; Hu et al., 2025; Zhao et al., 2025a). Holding a more stress-is-enhancing mindset has been found not only to buffer the detrimental effects of perceived stress on depression (Huebschmann & Sheets, 2020), anxiety (L. Zhang et al., 2026), hopelessness (Ming et al., 2021), and sleep duration (Zhao et al., 2025b), but also to foster positive psychological resources, including psychological resilience (Tuan, 2022), self-compassion (X. Zhang & Hao, 2025), and positive coping strategies (Caleon et al., 2023). Given these benefits and evidence that stress mindset is malleable (Ben-Avi et al., 2018; Crum et al., 2013), growing attention has been paid to identifying ways to promote a more stress-is-enhancing mindset.

Previous research has predominantly focused on how to foster a more stress-is-enhancing mindset (Crum et al., 2023; Laferton et al., 2023; X. Wang et al., 2026). For instance, Crum et al. (2017) demonstrated that a brief 3-min video emphasizing the cognitive benefits of stress facilitated the development of a more stress-is-enhancing mindset. Mansell et al. (2024) found that a 6-h face-to-face session about the positive consequences of stress promoted a more stress-is-enhancing mindset. While this body of work suggests that externally providing information about the beneficial consequences of stress can effectively foster a more stress-is-enhancing mindset, it remains unclear whether individuals can foster a more stress-is-enhancing mindset through internal cognitive strategies. Prior research has shown that individuals’ internal cognitive strategies can shape their mental representations of an entity (Epley et al., 2007), which in turn influence how they interpret that entity (F. Chen et al., 2020; Peng & Liu, 2025). Given that stress mindset reflects how individuals interpret the consequences of stress (Baynard-Montague & James, 2023; Sutin et al., 2023), internal cognitive strategies that alter individuals’ mental representations of stress may also shape their stress mindset. Therefore, examining whether and when internal cognitive strategies can foster a more stress-is-enhancing mindset is important for enriching the understanding of the antecedents of stress mindset.

To address this gap, the present research focuses on a specific internal cognitive strategy—anthropomorphism. Anthropomorphism refers to attributing humanlike characteristics to nonhuman entities (Epley et al., 2007; J. Huang et al., 2024). It is an inductive inference process in which individuals apply their knowledge about humans to reason about nonhuman entities, thereby representing these entities in human terms (Epley et al., 2008; Epley et al., 2007; Z. Zhang et al., 2023). As a result, individuals’ interpretations of anthropomorphized entities are influenced by their knowledge about humans that they apply to those entities, including social beliefs relevant to human interaction (F. Chen et al., 2023; L. Wang et al., 2023). Consistent with this reasoning, research has shown that individuals with different social beliefs interpret the same anthropomorphized entity differently (Kim & McGill, 2011; Wan et al., 2017). Accordingly, when stress is anthropomorphized, individuals with different social beliefs would interpret stress differently. This theoretical account is expected to operate when social beliefs are cognitively accessible (Epley et al., 2007). One such social belief that may be applied to anthropomorphized stress is generalized trust (Kim & McGill, 2011), which reflects individuals’ expectations regarding others’ goodwill and benign intentions (Dong et al., 2024). We propose that, because individuals with high generalized trust hold positive expectations that others possess goodwill and benign intentions, anthropomorphism would lead them to apply these expectations to anthropomorphized stress and perceive it as possessing similar goodwill and benign intentions. As a result, they may infer that anthropomorphized stress has intentions to help them, leading to higher perceived altruistic motivation and, in turn, a more stress-is-enhancing mindset. In contrast, individuals with low generalized trust hold less positive expectations regarding others’ goodwill and benign intentions, and thus do not make these interpretations. Therefore, the present research aims to investigate whether anthropomorphism and generalized trust jointly shape individuals’ stress mindset and to explore its underlying mechanism, potentially offering new insights into the antecedents of stress mindset.

Our theoretical account differs from stress reappraisal theories in two important respects. First, whereas stress reappraisal involves interpreting one’s stress responses elicited by a specific stressor as resources for coping with that stressor (Bosshard & Gomez, 2024; Crum et al., 2020), our theoretical account proposes that anthropomorphism leads individuals to represent stress itself in human terms, whereby they apply their social beliefs to interpret stress (F. Chen et al., 2023; Epley et al., 2007). Second, whereas stress reappraisal specifies the direction of interpretation by encouraging individuals to interpret stress responses as coping resources (Hagger et al., 2020; Jamieson et al., 2010), our theoretical account proposes that the interpretation of stress is shaped by the social beliefs individuals apply to its anthropomorphic representation (Kim & McGill, 2011; Wan et al., 2017). Therefore, our theoretical account is conceptually distinct from stress reappraisal. Moreover, because stress reappraisal does not provide a theoretical basis for predicting that the interpretation of anthropomorphized stress is contingent on individuals’ social beliefs, it does not provide an alternative explanation for the proposed effects.

## 2. Literature Review and Hypothesis Development

### 2.1. Anthropomorphism

Anthropomorphism refers to the attribution of humanlike characteristics, such as emotions, motivations, intentions, and minds, to non-human entities (Epley et al., 2007; J. Huang et al., 2024). The previous literature has examined the anthropomorphism of many entities, such as animals (Yoon et al., 2025), mountains (Lin et al., 2024), products (F. Huang et al., 2020), brands (Li et al., 2026; Liu et al., 2022), robots (J. Huang & Liao, 2026), time (May & Monga, 2014), and money (L. Wang et al., 2023). This body of work has predominantly focused on entities external to the individual, whereas little attention has been paid to the anthropomorphism of entities internal to the individual (F. Chen et al., 2020). Extending this line of research, we focus on stress as a specific internal entity that can be anthropomorphized.

Stress refers to the experience of anticipating or encountering adversity while pursuing one’s goals (Crum et al., 2013). The idea of portraying stress as human is not novel. Anthropomorphized stress has appeared in books, poems, podcasts, and blogs (Adner, 2009; Reso, 2025), with examples including “Stress as a guest in our home” (Chan, 2026), “Stress hates your brain” (Smith, 2014), and “Stress will be your best friend” (Wolff et al., 2021). Furthermore, a pilot study in which participants rated the extent to which different entities were viewed as humanlike confirmed that stress can be readily anthropomorphized, at a level comparable to that observed for other nonhuman entities in prior research (see Appendix A for details).

Moreover, existing research has predominantly focused on how anthropomorphism shapes individuals’ judgments of anthropomorphized entities (L. Wang et al., 2023). This line of research suggests that because anthropomorphism leads individuals to apply social beliefs relevant to human interaction to anthropomorphized entities (Epley et al., 2007; Kwak et al., 2025), anthropomorphism and social beliefs jointly influence subsequent judgments of these entities (L. L. Huang et al., 2024). For example, Wan et al. (2017) found that individuals with a stronger beautiful-is-good belief indicated greater preference for a product with superior appearance when the product was anthropomorphized than when it was not. Kim and McGill (2011) demonstrated that individuals with high power applied their feelings of social power over others to anthropomorphized entities, thereby perceiving them as less risky, whereas individuals with low power applied their feelings of social powerlessness to these entities, thereby perceiving them as more risky. While this body of literature has primarily examined social beliefs based on specific social cues (e.g., appearance and relative social power), less is known about whether anthropomorphism and social beliefs that are independent of external social cues, such as generalized trust, jointly influence individuals’ judgments of anthropomorphized entities. Extending this body of work, the present research investigates whether anthropomorphism and generalized trust jointly shape individuals’ stress mindset.

### 2.2. Anthropomorphism, Generalized Trust, Perceived Altruistic Motivation, and Stress Mindset

Generalized trust refers to a social belief reflecting the expectation of goodwill or benign intent from others in general (Dong et al., 2024). Previous research has shown that generalized trust shapes individuals’ perceptions of others (Muethel & Bond, 2013; Olivera-La Rosa et al., 2020). For example, Meng et al. (2022) found that individuals with higher generalized trust formed a more positive first impression of strangers. Muethel and Bond (2013) demonstrated that individuals’ generalized trust positively predicted perceptions of out-group members’ trustworthiness. While this line of research predominantly examines the role of generalized trust within human interactions, it remains unclear whether individuals apply this belief to understand nonhuman entities that are represented in human terms (i.e., anthropomorphized entities). To address this gap, the current research examines the joint effect of anthropomorphism and generalized trust on stress mindset. We argue that among individuals with high, but not low, generalized trust, anthropomorphizing stress leads them to perceive stress as having more altruistic motivation, which in turn promotes a more stress-is-enhancing mindset.

Perceived altruistic motivation refers to the perception that an agent is motivated to help, care for, and benefit others (Guo et al., 2025; Li et al., 2025). We propose that anthropomorphism leads to higher perceived altruistic motivation of stress among individuals with high generalized trust, but not among those with low generalized trust. Specifically, previous research suggests that individuals may apply their social beliefs, including generalized trust, to anthropomorphized non-human entities (Epley et al., 2007; Kim & McGill, 2011). Accordingly, when individuals anthropomorphize stress, those with high generalized trust may apply their positive expectations that others possess goodwill and benign intentions to anthropomorphized stress, and therefore perceive it as possessing similar goodwill and benign intentions. Because individuals tend to infer that agents perceived as possessing goodwill and benign intentions are motivated to help and benefit others (Gershon & Cryder, 2018; Mayer et al., 1995; Sutherland et al., 2016), those with high generalized trust may consequently perceive anthropomorphized stress as motivated to help and benefit them, leading to higher perceived altruistic motivation. In contrast, individuals with low generalized trust hold less positive expectations regarding others’ goodwill and benign intentions. Consequently, after anthropomorphizing stress, they may apply their less positive expectations to stress, making them less likely to perceive stress as having greater altruistic motivation. Taken together, anthropomorphizing stress is expected to increase perceived altruistic motivation among individuals with high, but not low, generalized trust.

Furthermore, the perceived altruistic motivation of stress may shape individuals’ stress mindset. Specifically, prior research has shown that perceiving an agent as having greater altruistic motivation may lead individuals to perceive the agent as more likely to produce beneficial outcomes (Chernev & Blair, 2015). For example, Gray (2012) found that the more individuals perceived others as having altruistic motivation, the more enjoyable they perceived the snacks prepared by those others. Based on this, we suggest that individuals who perceive stress as having more altruistic motivation are more likely to believe that stress would produce beneficial consequences for them, thereby holding a more stress-is-enhancing mindset. Therefore, the following hypotheses are proposed:

**H1.**  *Anthropomorphism and generalized trust jointly influence individuals’ stress mindset. Specifically, anthropomorphism (vs. non-anthropomorphism) leads to a more stress-is-enhancing mindset among individuals with high generalized trust, but not among those with low generalized trust.*

**H2.**  *Perceived altruistic motivation mediates the joint effect of anthropomorphism and generalized trust on stress mindset.*

### 2.3. Overview of the Current Research

Two studies were conducted to investigate the joint effect of anthropomorphism and generalized trust on stress mindset, as well as the mediating role of perceived altruistic motivation. Study 1 manipulated anthropomorphism in the context of general stress and examined whether anthropomorphism and generalized trust jointly shape individuals’ stress mindset. Study 2 replicated the findings of Study 1 in the context of a specific stressor and further examined whether perceived altruistic motivation serves as the underlying mechanism of this joint effect.

## 3. Study 1

Study 1 aimed to examine whether anthropomorphism and generalized trust jointly shape individuals’ stress mindset. Specifically, we predicted that anthropomorphism (vs. non-anthropomorphism) would promote a more stress-is-enhancing mindset among individuals with high generalized trust, but not among those with low generalized trust (H1).

### 3.1. Methods

#### 3.1.1. Participants and Design

Study 1 employed a 2 (anthropomorphism: anthropomorphism vs. non-anthropomorphism) × generalized trust (continuous) mixed-subjects design. Generalized trust was treated as a continuous moderator because it is conceptualized as a continuous trait (Dong et al., 2024; Yamagishi & Yamagishi, 1994). Retaining it in its original metric avoids the loss of information about individual differences and provides more informative and generalizable estimates of the proposed joint effect (Preacher, 2015; Preacher et al., 2005). This approach is also consistent with previous anthropomorphism research that examined moderation with a continuous moderator (J. Huang & Wang, 2023; Sheehan et al., 2020).

An a priori power analysis with G*Power 3.1 indicated that 74 participants were needed for 95% power, *α* = 0.05, and a medium effect size *f*^2^ = 0.15 (Lakens, 2013). Because no directly relevant empirical effect size estimates were available for the joint effect examined in the present study, a medium effect size was assumed based on previous anthropomorphism research that used an experimental design similar to that of the present study, involving an experimental manipulation of anthropomorphism and moderation by a continuous variable (Sheehan et al., 2020), as well as the common practice in psychological research when directly relevant empirical effect size estimates are unavailable (Bakker et al., 2020; Nijmeijer et al., 2021). We recruited 152 undergraduates (28 female) aged 17–23 years (*M*_age_ = 18.60, *SD* = 0.79) offline from a college in China. We programmed and administered the study using Qualtrics. All questions were set to require a mandatory response, resulting in no missing data. The study took approximately 6 min to complete. Participants received course credit for their participation. No attention check items were included, and no participants were excluded from the analyses. The study gained approval from the second author’s University Ethics Committee.

#### 3.1.2. Materials

##### Manipulation of Anthropomorphism

The anthropomorphism manipulation was adapted from Hur et al. (2015). Participants were randomly assigned to either an anthropomorphism condition or a non-anthropomorphism condition. In the anthropomorphism condition, participants were instructed to imagine that stress had come to life as a person and describe what kind of person he or she would be in terms of appearance, personality, and occupation. In the non-anthropomorphism condition, participants were asked to describe the features and characteristics of stress. A pretest confirmed the validity of the anthropomorphism manipulation. A total of 120 participants (77 female; aged 19–64 years, *M*_age_ = 33.22, *SD* = 11.35) recruited from Credamo, a widely used Chinese online survey platform (www.credamo.com (accessed on 12 June 2026); Pan et al., 2024; Ye & Li, 2025), were randomly assigned to the anthropomorphism condition or non-anthropomorphism condition. They then rated their perceived anthropomorphism by two items on a 7-point Likert scale (i.e., “stress looks like a person”; “stress has its own personality”; 1 = strongly disagree, 7 = strongly agree; *r* = 0.86, *p* < 0.001; Kim et al., 2016). Results showed that participants in the anthropomorphism condition (*M* = 5.70, *SD* = 1.20) reported significantly higher perceived anthropomorphism than those in the non-anthropomorphism condition (*M* = 3.93, *SD* = 1.73), *t* = 6.50, *p* < 0.001, *Cohen’s d* = 1.19.

##### Measurement of Generalized Trust

Generalized trust was measured using a 5-item 7-point scale from Horita and Yamazaki (2022) (e.g., “Most people are basically good-natured and kind”; 1 = strongly disagree, 7 = strongly agree; *α* = 0.86). Higher mean scores on this scale indicated greater generalized trust.

##### Measurement of Stress Mindset

Stress mindset was assessed using an 8-item 7-point scale from Crum et al. (2013) (e.g., “The effects of stress are positive and should be utilized”; 1 = strongly disagree, 7 = strongly agree; four items were reverse-coded; *α* = 0.69). To further examine the construct validity of this scale, a single-factor confirmatory factor analysis was conducted. The fit indices (χ^2^/*df* = 1.75, TLI = 0.94, CFI = 0.97, RMSEA = 0.07, SRMR = 0.05) supported the construct validity of the stress mindset measure. Higher mean scores represented a more stress-is-enhancing mindset.

#### 3.1.3. Procedures

After providing informed consent, participants were randomly assigned to either an anthropomorphism condition or a non-anthropomorphism condition. Participants then completed measures of stress mindset and generalized trust. Next, they rated their perceived anthropomorphism as a manipulation check. Finally, participants reported their demographic information.

### 3.2. Results

Descriptive statistics for all variables are presented in Table A2 (see Appendix B).

**Figure 1:**
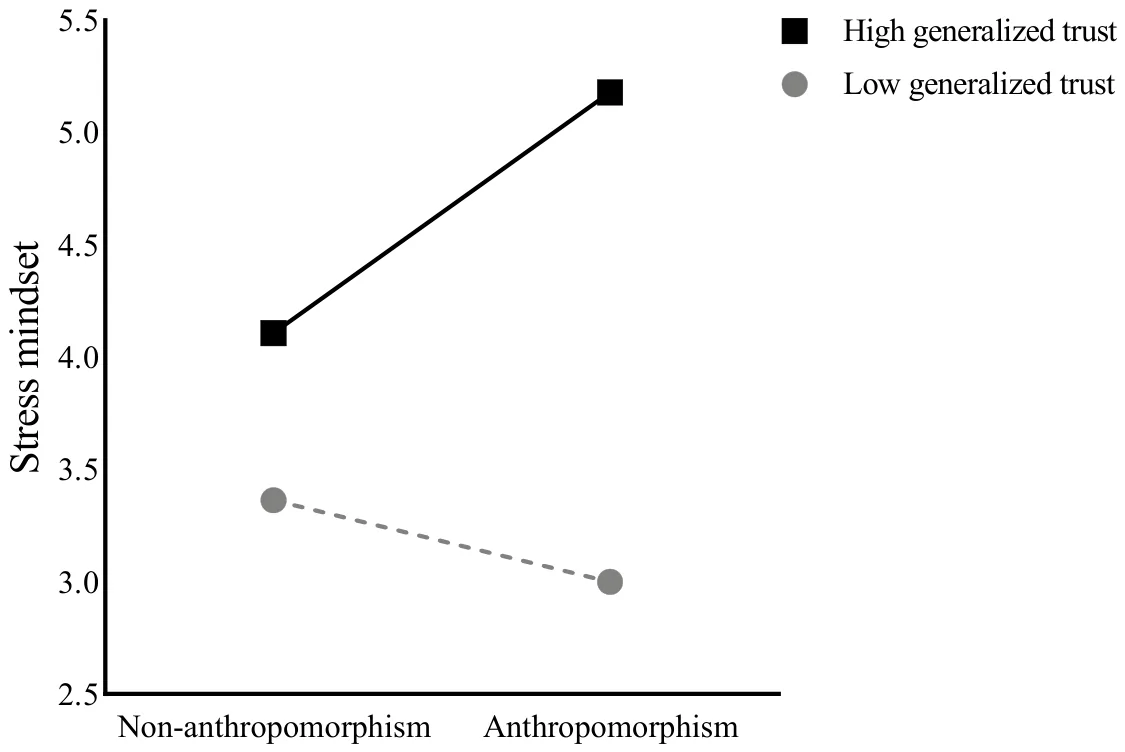
The joint effect of anthropomorphism and generalized trust on stress mindset. Note. Higher stress mindset scores indicate a more stress-is-enhancing mindset.

A moderation analysis was conducted using the PROCESS macro (Model 1; 5000 bootstrap samples), with anthropomorphism as the independent variable (coded as non-anthropomorphism = 0, anthropomorphism = 1), mean-centered generalized trust as the moderator, and stress mindset as the dependent variable. Results revealed no main effects of anthropomorphism (*β* = 0.08, *SE* = 0.12, 95% CI = [−0.157, 0.315]) or generalized trust (*β* = −0.05, *SE* = 0.09, 95% CI = [−0.225, 0.120]), but a significant interaction between anthropomorphism and generalized trust (*β* = 0.30, *SE* = 0.12, 95% CI = [0.053, 0.538]) (see Figure 1). The interaction term explained 4% additional variance in stress mindset (Δ*R*^2^ = 0.04, *F* = 5.79, *p* = 0.02). Simple slope analysis revealed that, for individuals with high generalized trust (+1 *SD*), those in the anthropomorphism condition reported higher stress mindset than those in the non-anthropomorphism condition (*β* = 0.37, *SE* = 0.17, 95% CI = [0.033, 0.701]). For individuals with low generalized trust (−1 *SD*), no significant difference was found between the two conditions (*β* = −0.21, *SE* = 0.17, 95% CI = [−0.544, 0.125]). These results support H1.

To further validate the robustness of our findings, we conducted two supplementary analyses. First, a moderated analysis was conducted using the PROCESS macro (Model 1; 5000 bootstrap samples) with heteroscedasticity-consistent standard errors (HC3). Results confirmed that the interaction between anthropomorphism and generalized trust remained significant (*β* = 0.30, *SE* = 0.14, 95% CI = [0.012, 0.578]). Second, to account for measurement error, we conducted latent variable structural equation modeling (SEM; 5000 bootstrap samples), using the item-to-construct balance approach to create three parcels for the stress mindset construct, thereby reducing potential parameter estimation bias associated with modeling individual items in a relatively small sample (Little et al., 2002; Wu & Wen, 2011). The measurement model showed acceptable fit (χ^2^/*df* = 1.59, TLI = 0.96, CFI = 0.97, RMSEA = 0.06, SRMR = 0.05), and the interaction between anthropomorphism and generalized trust remained significant (*β* = 0.19, *SE* = 0.09, *p* = 0.02, 95% CI = [0.028, 0.360]). These findings support the robustness of the observed moderation effect.

### 3.3. Discussion

The results of Study 1 confirmed H1 by demonstrating that anthropomorphism (vs. non-anthropomorphism) led to a more stress-is-enhancing mindset among individuals with high, but not low, generalized trust. Study 1 has two limitations. First, Study 1 examined the proposed joint effect in the context of general stress. While this approach is reasonable and commonly used for investigating the antecedents of stress mindset (Crum et al., 2013; Sutin et al., 2023), it remains unclear whether anthropomorphism and generalized trust also jointly shape stress mindset in the context of a specific stressor. Specifically, previous research has distinguished between stress mindset in general and stress mindset in the context of a specific stressor (Crum et al., 2013) and has reported inconsistent findings regarding the association between them (Jenkins et al., 2021), suggesting that stress mindset regarding stress in general may not necessarily correspond to stress mindset regarding a specific stressor. Because previous research suggests that anthropomorphism is an inductive inference process through which individuals represent nonhuman entities in human terms (Epley et al., 2008; Epley et al., 2007), anthropomorphizing stress is expected to lead individuals to apply social beliefs, such as generalized trust, when interpreting stress regardless of whether it is stress in general or stress elicited by a specific stressor. Accordingly, the joint effect of anthropomorphism and generalized trust on stress mindset is expected to be observed in the context of a specific stressor. Therefore, Study 2 examined whether anthropomorphism and generalized trust jointly shape stress mindset in the context of a specific stressor, thereby testing the generalizability of our findings. Second, the underlying mechanism driving this joint effect has not yet been explored. To address this issue, Study 2 investigated whether perceived altruistic motivation mediates the joint effect of anthropomorphism and generalized trust on stress mindset.

## 4. Study 2

The purpose of Study 2 was to replicate the findings of Study 1 in the context of a specific stressor, thereby enhancing the generalizability of our findings, and to further examine the mechanism underlying the joint effect of anthropomorphism and generalized trust on stress mindset. We predicted that perceived altruistic motivation would mediate the joint effect of anthropomorphism and generalized trust on stress mindset, such that anthropomorphism (vs. non-anthropomorphism) would increase perceived altruistic motivation, which in turn would lead to a more stress-is-enhancing mindset among individuals with high generalized trust, but not among those with low generalized trust (H2).

### 4.1. Methods

#### 4.1.1. Participants and Design

Study 2 employed a 2 (anthropomorphism: anthropomorphism vs. non-anthropomorphism) × generalized trust (continuous) mixed-subjects design. An a priori power analysis with G*Power indicated that 74 participants were needed for 95% power, *α* = 0.05, and a medium effect size *f*^2^ = 0.15 (Lakens, 2013). The same rationale for assuming a medium effect size as in Study 1 was adopted. We recruited 240 adults (169 female) aged 19–69 years (*M*_age_ = 32.08, *SD* = 9.18) from Credamo, a widely used Chinese online survey platform (Pan et al., 2024; Ye & Li, 2025). We programmed and administered the study using Credamo. All questions were set to require a mandatory response, resulting in no missing data. The study took approximately 7 min to complete. Participants received 1 RMB for their participation. One attention check item was included (i.e., “Please choose the fourth option regardless of your preferences”) (S. Chen et al., 2024). All participants passed the attention check and were therefore included in the analyses. The study gained approval from the second author’s University Ethics Committee.

#### 4.1.2. Materials

##### Manipulation of Anthropomorphism

To test the proposed effect in the context of a specific stressor, the manipulation of anthropomorphism was similar to that used in Study 1, except that it was embedded within a specific stressful scenario rather than general stress. Because job stressors are among the most common stressors encountered by adults worldwide and are also prevalent among Chinese adults (Gallup, 2026; Yan et al., 2022; Zang et al., 2022), the population from which the present sample was drawn, Study 2 adopted a job stress scenario to enhance the ecological validity of the study. Specifically, following Ben-Avi et al. (2018), participants were first asked to imagine themselves as an employee named Ben who was experiencing high levels of stress due to a heavy workload, including managerial responsibilities, working long hours, and multitasking. These job stressors have been identified in previous research as common sources of job stress among Chinese adults (Wei et al., 2023; Q. Zhang & Zhao, 2025; S. Zhang et al., 2023), further supporting the cultural relevance of the scenario. Participants then described the stress they experienced. Next, participants were randomly assigned to either an anthropomorphism condition or a non-anthropomorphism condition. In the anthropomorphism condition, participants were instructed to imagine that the stress they experienced in the preceding scenario had come to life as a person and describe what kind of person he or she would be in terms of appearance, personality, and occupation. In the non-anthropomorphism condition, participants were asked to describe the features and characteristics of the stress they experienced in the scenario. To assess the effectiveness of the manipulation, participants completed the same manipulation-check items used in Study 1, with the items referring specifically to the stress experienced in the preceding scenario (*r* = 0.79, *p* < 0.001).

##### Measurement of Generalized Trust

Generalized trust was measured using the same scale as in Study 1 (*α* = 0.90).

##### Measurement of Stress Mindset

Stress mindset was measured using the same items as in Study 1, adapted to refer specifically to the stress experienced in the preceding scenario (*α* = 0.90).

##### Measurement of Perceived Altruistic Motivation

Perceived altruistic motivation was assessed using a 3-item 7-point scale adapted from Li et al. (2025) (e.g., “The aforementioned stress wants to help me”; 1 = strongly disagree, 7 = strongly agree; *α* = 0.82). Higher mean scores indicated greater perceived altruistic motivation of the stress experienced in the scenario.

#### 4.1.3. Procedures

The procedure of Study 2 was identical to Study 1, except that an additional measure of perceived altruistic motivation was included after the stress mindset measure.

### 4.2. Results

#### 4.2.1. Manipulation Check

As expected, participants in the anthropomorphism condition (*M* = 4.49, *SD* = 1.34) reported significantly higher perceived anthropomorphism than those in the non-anthropomorphism condition (*M* = 3.64, *SD* = 1.59), *t* = 4.48, *p* < 0.001, *Cohen’s d* = 0.58.

#### 4.2.2. Regression Diagnostics

Regression diagnostics indicated no concerns regarding multicollinearity or influential observations. Specifically, VIFs ranged from 1.00 to 1.12, and Cook’s distances ranged from 0.00 to 0.13. In addition, the residuals did not significantly deviate from normality (Kolmogorov–Smirnov D = 0.05, *p* > 0.200).

#### 4.2.3. Joint Effect of Anthropomorphism and Generalized Trust on Stress Mindset

Descriptive statistics for all variables are presented in Table A3 (see Appendix C).

A moderation analysis was conducted using the PROCESS macro (Model 1; 5000 bootstrap samples), with anthropomorphism as the independent variable (coded as non-anthropomorphism = 0, anthropomorphism = 1), mean-centered generalized trust as the moderator, and stress mindset as the dependent variable. Results revealed no main effect of anthropomorphism (*β* = 0.10, *SE* = 0.14, 95% CI = [−0.186, 0.384]), but a significant main effect of generalized trust (*β* = 0.23, *SE* = 0.10, 95% CI = [0.033, 0.426]) and a significant interaction between anthropomorphism and generalized trust (*β* = 0.32, *SE* = 0.15, 95% CI = [0.025, 0.609]). The interaction term explained 2% additional variance in stress mindset (Δ*R*^2^ = 0.02, *F* = 4.58, *p* = 0.03). Simple slope analysis revealed that, for individuals with high generalized trust (+1 *SD*), those in the anthropomorphism condition reported higher stress mindset than those in the non-anthropomorphism condition (*β* = 0.41, *SE* = 0.21, 95% CI = [0.002, 0.811]). For individuals with low generalized trust (−1 *SD*), no significant difference was found between the two conditions (*β* = −0.22, *SE* = 0.21, 95% CI = [−0.620, 0.187]). These findings support H1 again.

To further validate the robustness of our findings, we conducted the same two supplementary analyses as in Study 1. First, a moderated analysis was conducted using the PROCESS macro (Model 1; 5000 bootstrap samples) with heteroscedasticity-consistent standard errors (HC3). Results confirmed that the interaction between anthropomorphism and generalized trust remained significant (*β* = 0.32, *SE* = 0.15, 95% CI = [0.031, 0.603]). Second, latent variable structural equation modeling (SEM; 5000 bootstrap samples) yielded acceptable model fit (χ^2^/*df* = 1.99, TLI = 0.97, CFI = 0.98, RMSEA = 0.06, SRMR = 0.03), and the interaction between anthropomorphism and generalized trust remained significant (*β* = 0.16, *SE* = 0.07, *p* = 0.01, 95% CI = [0.035, 0.292]). These findings support the robustness of the observed moderation effect.

#### 4.2.4. Mediating Role of Perceived Altruistic Motivation

**Figure 2:**
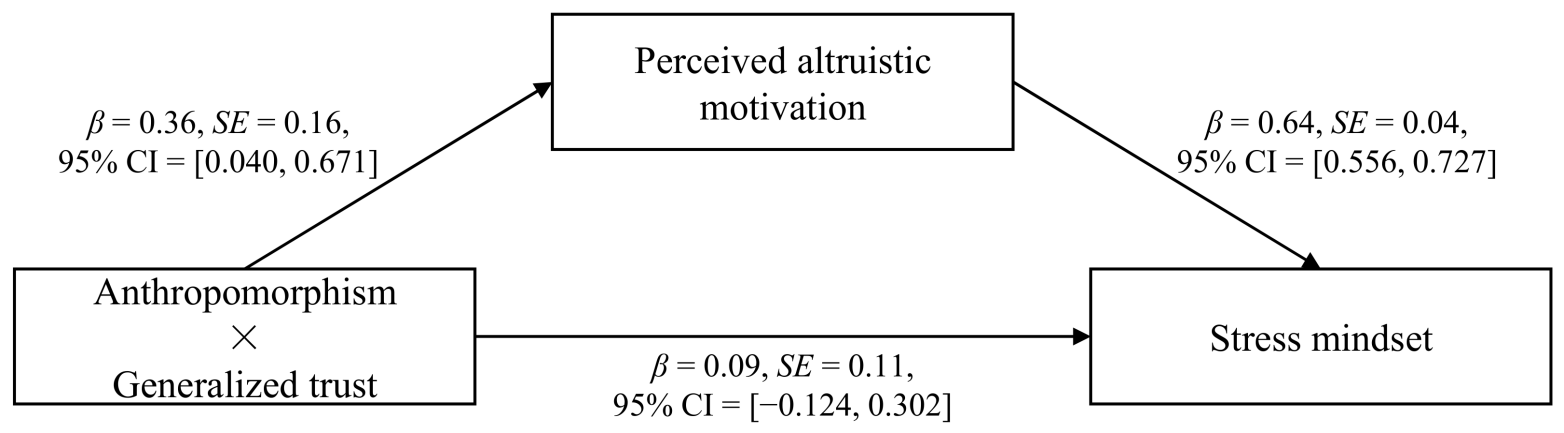
The mediating role of perceived altruistic motivation in the joint effect of anthropomorphism and generalized trust on stress mindset.

To further test the proposed mediating role of perceived altruistic motivation, a moderated mediation analysis was conducted using the PROCESS macro (Model 8; 5000 bootstrap samples), with anthropomorphism as the independent variable (coded as non-anthropomorphism = 0, anthropomorphism = 1), mean-centered generalized trust as the moderator, perceived altruistic motivation as the mediator and stress mindset as the dependent variable. Results indicated a significant index of moderated mediation (*β* = 0.23, *SE* = 0.10, 95% CI = [0.026, 0.433]) (see Figure 2). The interaction term explained 2% additional variance in perceived altruistic motivation (Δ*R*^2^ = 0.02, *F* = 4.93, *p* = 0.03). Simple slope analysis revealed that, for individuals with high generalized trust (+1 *SD*), the mediating effect was significant (*β* = 0.30, *SE* = 0.15, 95% CI = [0.010, 0.593]). For individuals with low generalized trust (−1 *SD*), the mediating effect was not significant (*β* = −0.15, *SE* = 0.14, 95% CI = [−0.415, 0.117]). These results support H2.

To further validate the robustness of our findings, we conducted the same two supplementary analyses as in Study 1. First, a moderated mediation analysis was conducted using the PROCESS macro (Model 8; 5000 bootstrap samples) with heteroscedasticity-consistent standard errors (HC3). Results confirmed that the index of moderated mediation remained significant (*β* = 0.23, *SE* = 0.11, 95% CI = [0.023, 0.440]). Second, latent variable structural equation modeling (SEM; 5000 bootstrap samples) yielded acceptable model fit (χ^2^/*df* = 2.02, TLI = 0.97, CFI = 0.97, RMSEA = 0.07, SRMR = 0.04), and the index of moderated mediation was marginally significant (*β* = 0.20, *SE* = 0.12, *p* = 0.09). Although the index of moderated mediation was only marginally significant in the SEM analysis, its direction and magnitude were consistent with those obtained in the primary analysis, providing partial support for the robustness of our findings.

### 4.3. Discussion

Study 2 not only replicated the joint effect of anthropomorphism and generalized trust on stress mindset in the context of a specific stressor, thereby reinforcing the robustness and generalizability of our findings, but also provided support for H2 by identifying perceived altruistic motivation as an underlying mechanism. Specifically, among individuals with high, but not low, generalized trust, anthropomorphism (vs. non-anthropomorphism) increased perceived altruistic motivation, which in turn led to a more stress-is-enhancing mindset.

## 5. General Discussion

The two experiments examined whether anthropomorphism and generalized trust jointly shape individuals’ stress mindset, and whether this joint effect is mediated by perceived altruistic motivation. Study 1 found that, compared with the non-anthropomorphism condition, anthropomorphism led individuals with high, but not low, generalized trust to have a more stress-is-enhancing mindset in the context of general stress. Study 2 extended these findings by replicating this joint effect in the context of a specific stressor and by demonstrating the mediating role of perceived altruistic motivation. Specifically, among individuals with high generalized trust, anthropomorphism increased perceived altruistic motivation, which in turn led to a more stress-is-enhancing mindset. In contrast, this indirect effect was not observed among individuals with low generalized trust.

### 5.1. Theoretical Contributions

The current work makes several contributions to the existing literature. First, it adds to the literature on anthropomorphism in two ways. On one hand, previous research has primarily focused on the anthropomorphism of entities external to individuals, such as products (F. Huang et al., 2020), brands (Liu et al., 2022), robots (J. Huang & Liao, 2026), time (May & Monga, 2014), and money (L. Wang et al., 2023). The present research extends this work by demonstrating that anthropomorphism can also be applied to an internal experience—stress—and that anthropomorphizing stress can promote a more stress-is-enhancing mindset. Thus, the present work broadens the scope of anthropomorphism research by identifying stress as a novel anthropomorphized entity. On the other hand, prior research has examined how anthropomorphism and social beliefs based on specific social cues (e.g., appearance and relative social power) jointly shape individuals’ judgments of anthropomorphized entities (Kim & McGill, 2011; Wan et al., 2017). The present research advances this line of work by demonstrating that anthropomorphism and generalized trust—a social belief that does not depend on specific external social cues—jointly influence individuals’ stress mindset. More broadly, our findings suggest that future research may benefit from examining how anthropomorphism and social beliefs that are independent of specific social cues jointly shape individuals’ judgments of anthropomorphized entities.

Second, this research extends the stress mindset literature by broadening our understanding of its antecedents. Previous research has primarily shown that externally provided persuasive information about the beneficial consequences of stress can promote a more stress-is-enhancing mindset (Crum et al., 2017; Mansell et al., 2024). Our work extends this body of research by showing that an internal cognitive strategy—anthropomorphism—can also jointly shape individuals’ stress mindset with generalized trust. This finding suggests that stress mindset is shaped not only by externally provided persuasive information but also by the interplay between individuals’ internal cognitive strategies and social beliefs.

Third, we identify perceived altruistic motivation as the mechanism underlying the joint effect of anthropomorphism and generalized trust on stress mindset. Diverging from previous research, which has primarily explored how characteristics of agents, such as power (L. Huang et al., 2026), warmth (Gershon & Cryder, 2018), and intimate self-disclosures (Li et al., 2025) shape individuals’ perceived altruistic motivation toward them, the present research highlights the role of perceivers’ internal cognitive strategies and social beliefs. Furthermore, our work connects three areas in the literature that have evolved largely in parallel: anthropomorphism (Epley et al., 2007; J. Huang et al., 2024), generalized trust (Dong et al., 2024; Meng et al., 2022), and stress mindset (Caleon et al., 2023; Crum et al., 2013; L. Zhang et al., 2026).

### 5.2. Practical Implications

The present research suggests that anthropomorphizing stress may be a promising strategy for fostering a more stress-is-enhancing mindset, but its effectiveness is contingent on individuals’ generalized trust. Specifically, anthropomorphizing stress may encourage a more stress-is-enhancing mindset among individuals with high generalized trust, whereas it did not produce the same effect among individuals with low generalized trust and showed a nonsignificant trend toward a less stress-is-enhancing mindset than the non-anthropomorphism condition. Accordingly, practitioners should first assess individuals’ generalized trust using established and validated generalized trust scales and use the resulting scores to gauge individuals’ relative level of generalized trust within the target population. Based on this assessment, practitioners may determine, on an individual basis, whether anthropomorphizing stress is an appropriate intervention. For individuals with high generalized trust, practitioners may encourage them to anthropomorphize stress through intervention activities such as expressive writing exercises in which individuals imagine stress as a person and describe its appearance, personality, and occupation. For individuals with low generalized trust, practitioners may instead consider alternative interventions to foster a stress-is-enhancing mindset rather than anthropomorphizing stress. Furthermore, because our findings indicate that individuals are more likely to hold a stress-is-enhancing mindset when they perceive stress as having higher altruistic motivation, practitioners may promote a more stress-is-enhancing mindset by guiding individuals to anthropomorphize stress in ways that emphasize its motivation to help and benefit them.

### 5.3. Limitations and Future Research

This research has several limitations. First, the samples in the present research were gender imbalanced, which may limit the generalizability of our findings. Although supplementary analyses showed that the proposed effects remained significant after controlling for gender (joint effect in Study 1: *β* = 0.29, *SE* = 0.12, 95% CI = [0.049, 0.537]; joint effect in Study 2: *β* = 0.33, *SE* = 0.15, 95% CI = [0.034, 0.617]; index of moderated mediation in Study 2: *β* = 0.23, *SE* = 0.11, 95% CI = [0.026, 0.444]), future research using gender-balanced samples is needed to test the generalizability of our findings and examine whether the observed effects are consistent across gender.

Second, we examined the proposed moderated mediation model in Study 2 by measuring the mediator and outcome in a single session, which limits the strength of the causal inferences that can be drawn because common method bias and temporal ambiguity cannot be fully ruled out. A supplementary analysis using an unmeasured latent method construct (ULMC) indicated that adding a common method bias (CMB) factor did not meaningfully improve the fit of the hypothesized three-factor model (baseline model: χ^2^/*df* = 2.42, TLI = 0.93, CFI = 0.94, RMSEA = 0.08, SRMR = 0.05; ULMC model: χ^2^/*df* = 2.44, TLI = 0.93, CFI = 0.94, RMSEA = 0.08, SRMR = 0.05), suggesting that common method bias is unlikely to be a serious concern in the present study (Podsakoff et al., 2024). Nevertheless, the significant index of moderated mediation should be interpreted with caution until these findings are replicated using longitudinal experimental designs to further reduce concerns regarding common method bias and temporal ambiguity (Voigt & Strauss, 2024).

Third, consistent with previous anthropomorphism research, we manipulated anthropomorphism by asking participants to imagine that stress had come to life as a person, thereby inducing an anthropomorphic representation of stress (F. Chen et al., 2020; Hur et al., 2015; Mishra et al., 2026). However, it is important to recognize that anthropomorphism can also be induced by presenting visual or verbal anthropomorphic cues, such as images with human-like elements (e.g., eyes and limbs), speech bubbles, and first-person self-introductions (W. H. Huang, 2026; F. Huang et al., 2020). Given that these two approaches may involve different cognitive processes (Karpinska-Krakowiak, 2025), future research could examine the robustness of our findings by manipulating stress anthropomorphism through visual or verbal anthropomorphic cues.

Fourth, this research asked participants to anthropomorphize stress without assigning it a specific role. However, prior research suggests that individuals can anthropomorphize non-human entities in different roles, such as servants (Lv et al., 2025), partners (L. Wang & Touré-Tillery, 2025), controllers (Puzakova & Aggarwal, 2018), or a person dependent on them (Mishra et al., 2026), and that these roles shape individuals’ judgments of those entities (Teng et al., 2024; Wu & Jiang, 2019). As such, future research could examine whether the role assigned to anthropomorphized stress moderates the joint effect of anthropomorphism and generalized trust on stress mindset.

Finally, although the current research demonstrates a short-term joint effect of anthropomorphism and generalized trust on stress mindset, it remains unclear whether this joint effect strengthens or weakens as individuals repeatedly anthropomorphize stress. Hence, an important direction for future research would be to examine whether and how this joint effect changes as individuals repeatedly anthropomorphize stress. In addition, future research could investigate whether stress-related factors, such as the type (e.g., academic versus life stress), magnitude, and frequency of stress, moderate this joint effect.

## 6. Conclusions

The present findings suggest that anthropomorphizing stress fosters a stress-is-enhancing mindset among individuals with high, but not low, generalized trust. This joint effect operates through increased perceived altruistic motivation. Theoretically, these findings provide new insights into the anthropomorphism literature by identifying stress as a novel anthropomorphized entity and suggesting that individuals may apply social beliefs that are independent of specific external social cues, such as generalized trust, when interpreting anthropomorphized stress. Moreover, this research highlights the importance of jointly considering internal cognitive strategies and social beliefs in explaining the antecedents of stress mindset. From a practical perspective, the present findings suggest that anthropomorphizing stress may serve as a promising strategy for fostering a more stress-is-enhancing mindset, but its effectiveness depends on individuals’ generalized trust.

## Data Availability

The data presented in this study are available on request from the corresponding authors due to privacy and ethical restrictions.

## Appendix A. Pilot Study: Anthropomorphism Tendency for Different Entities

To validate that the extent to which people anthropomorphize stress is comparable to that for other non-human entities, as observed in previous research, we conducted a pilot study following F. Chen et al. (2020).

### Appendix A.1. Methods

A total of 75 undergraduates (14 females) aged 17–22 years (*M*_age_ = 18.53, *SD* = 0.78) were recruited offline from a college in China (Pan et al., 2024). Participants reported their tendency to anthropomorphize stress and 14 additional non-human entities (i.e., money, sadness, dog, brand, time, cookie, car, insect, mountain, steam iron, battery, light bulb, phone, and robot), which had been used as anthropomorphic targets in prior research (F. Chen et al., 2020; May & Monga, 2014). Participants rated their tendency to anthropomorphize each entity using the same well-established 5-item anthropomorphism scale (e.g., “To what extent do you think of stress as having a will of its own?”; 1 = *not at all*, 7 = *very much*; Waytz et al., 2010) in a random order. The scale demonstrated high reliability across all entities (all *αs* > 0.93).

### Appendix A.2. Results

Results suggest that stress is more readily anthropomorphized than many of the entities examined. The tendency to anthropomorphize stress was significantly higher than all other tested entities (*p*s < 0.013) except for dog (significantly lower, *p* < 0.001), insect (significantly lower, *p* < 0.001), time (not significantly higher, *p* = 0.928), sadness (not significantly higher, *p* = 0.832), and robot (not significantly higher, *p* = 0.124; see Table A1 for details). Thus, findings from the pilot study suggest that stress can be readily anthropomorphized by individuals.

**Table A1:** Degree of anthropomorphism tendency for different entities (pilot study).

Anthropomorphic Entity	*M*	*SD*
Dog	5.77	1.46
Insect	4.78	1.67
Stress	3.17	1.95
Time	3.15	2.06
Sadness	3.13	1.83
Robot	2.76	1.66
Phone	2.58	1.87
Money	2.56	1.99
Brand	2.47	1.72
Mountain	2.35	1.53
Car	2.01	1.57
Light bulb	1.70	1.26
Batter	1.64	1.20
Cookie	1.52	1.03
Steam iron	1.46	0.89

## Appendix B. Descriptive Statistics of Variables in Study 1

**Table A2:** Descriptive statistics of variables in Study 1 (*N* = 152).

Variables	*M*	Mdn	Min	Max	*SD*	Percentage (%)
Anthropomorphism						
Anthropomorphism = 1						50.00
Non-anthropomorphism = 0						50.00
Generalized trust	4.13	4.00	1.80	7.00	0.98	
Stress mindset	4.26	4.13	1.25	7.00	0.74	

## Appendix C. Descriptive Statistics of Variables in Study 2

**Table A3:** Descriptive statistics of variables in Study 2 (*N* = 240).

Variables	*M*	Mdn	Min	Max	*SD*	Percentage (%)
Anthropomorphism						
Anthropomorphism = 1						50.00
Non-anthropomorphism = 0						50.00
Generalized trust	5.23	5.40	1.80	6.80	0.98	
Perceived altruistic motivation	3.78	3.83	1.00	6.67	1.27	
Stress mindset	3.72	3.63	1.00	6.38	1.18	
